# Service of Prof. Andrews in Mercy Hospital

**Published:** 1872-01-01

**Authors:** 


### Clinical Reports.

### SERVICE OF PROF. ANDREWS IN MERCY HOSPITAL.

#### MULBERRY CALCULUS.

A boy sixteen years of age was admitted with an irritation of the bladder, which had lasted since early childhood. The sound disclosed a calculus. After a few days, constitutional preparation, Dr. Andrews cut for the stone by the lateral method, and removed a fine mulberry calculus about an inch in diamter.

Second day. Patient passes the water by the natural route.

Third day. A little dribbling from the wound.

Fourth day. Water all passes *per vias naturales.*

Tenth day. Discharged cured.

#### PHOSPHATIC CALCULUS.

This patient, a boy seven years of age, was admitted with great pain and vesical tenesmus. The sound disclosed a calculus, which was removed by the same method as in the previous patient. It proved to be a large stone of the phosphatic variety. The patient was placed upon the use of tincture of iron as a safeguard against pyaemia, and discharged cured on the fifteenth day.

#### CURIOUS OPIUM CURE.

A patient entered for tertiary syphilis. He was found to be an opium eater, and to be in process of cure by a quack method of considerable ingenuity. He was furnished with a pint bottle of medicine, intended to last him a month, and for which he paid ten dollars. He was assured that it would prove a perfect substitute for his opiate, so that he could leave off the latter at once without distress. This he found to be the case, and very naturally, for the bottle appeared to contain a solution of morphine colored with some deep red dye. Every month he sends ten dollars for a new bottle, which the quack makes a little weaker than its predecessor; and at length, in about a year and a half, he gets "tapered off" to nothing, and is cured. This patient is now on his thirteenth bottle. Fearing he would not persevere in reform, Dr. Andrews refrained from exposing the deception, but advised him when he sent for the next bottle to "taper it off" himself a little faster by putting into the bottle after every dose, as much pure water as the bulk of the dose taken out.

#### COMPOUND DISLOCATION OF THE ELBOW. ANTISEPTIC TREATMENT. (OUTSIDE PATIENT.)

A woman was thrown from a vehicle, tearing loose all the ligaments of the left elbow, so that the bones could be moved in all directions. A large wound existed in front, through which the finger could be inserted freely into the joint. Dr. Andrews drenched the interior with carbolic acid and water, fifteen grains to the ounce, and covered the wound with lint dipped in carbolic acid and castor oil, forty grains to the ounce. This dressing and injection was daily repeated. Arm laid in a tin splint.

Fourteenth day after injury. No anchylosis, nor even synovitis.

Sixteenth day. Sharp attack of synovitis, which was combated with ice-bags, and continued use of the carbolated water and carbolated oil.

Twenty-second day. Synovitis subdued.

Thirty-fourth day. Two cellular abcesses, but no anchylosis.

Fortieth day. No anchylosis.

Fifty-sixth day. Wound healed. No anchylosis of the joint, and the case promises a splendid success. There was scarcely a spoonful of pus discharged during the whole treatment.

COMPOUND FRACTURE NEAR ELBOW.

Boy run over by a wagon, crushing off the humerus close to the joint. Extensive wounds. Ends of fragments denuded of periosteum. Gave ether, and trimmed off the denuded ends of the bone. Drenched the interior with carbolated water as in the other case, and covered the wounds with lint dipped in the carbolated oil. Tinct. iron internally, and repeat the dressing and injections once a day. Tin right angular splint.

Fourth day. Doing finely. No inflammation, and scarcely any pus. Continue.

No inflamation followed, and now at the twentieth day, the wound is rapidly healing.

SINGULAR MALFORMATION.

Dr. Chas. Badger, of Milton, Wis., sent in a model of the bones of the left arm of a patient, presenting a remarkable deviation from the usual anatomy. This limb has two elbows and two forearms, one on the end of the other. The humerus is only about four or five inches long, and terminates at the upper elbow. From this articulation two bones, one behind the other, extend down to the normal elbow. The posterior bone slides up and down on the anterior one when the lower elbow is extended and flexed. From the lower elbow to the extremity of the limb the usual anatomy prevails. The motion of the upper elbow is quite limited. The right arm is perfectly normal, but of late years rather weak, the patient being old, so that the abnormal arm is at present much the best one. Those scientists who see in all human monstrosities a reversion to the type of some inferior animal, will now have to bestir themselves to hunt up some animal with two elbows on each arm. The specimen will be placed in the Chicago Medical College museum.

PENIS BATHS.

Prof. Andrews has been experimenting in cases of chancre, to see if long-continued baths of the penis in astringent solutions weak enough to avoid smarting, will have the same curative effect as the brief and painful cautery of nitric acid. One case especially, where a phagedenic chancre had continued to spread in spite of repeated cauteries with the acid, was promptly arrested by the penis baths. For this purpose Dr. A. uses a small jar holding a pint. This is filled with a solution containing cryst. carbolic acid, five grains to the ounce, and chloride of zinc, two grains to the ounce of water. The patient, either sitting or lying, lays the penis in the bath for one hour three times a day. The strength of the solution varies according to the sensitiveness of the patient, being kept in all cases just below the smarting point.

In cases of gonorrhœa, the Doctor uses the same solution by way of irrigation. He has a reservoir holding a quart or more, five feet above the patient's penis. A rubber tube, terminating in a flexible catheter, size No. 6, is attached, and the catheter inserted into the uthrea, but not into the bladder. A quart of the solution is placed in the reservoir and allowed to run into the urethra, where escaping from the eye of the catheter, it runs out again between the catheter and the walls of the canal, giving the latter a thorough and long-continued drenching. The effect is excellent.

PRESENTIMENTS OF DEATH.

On this subject Dr. Andrews remarked as follows:

Where a patient, without being frightened, excited, nor yet impressed by being told of danger by others, coolly arrives at the opinion that he will die, it is a serious symptom. I do not think the populor opinion is correct which asserts that in such cases the patient dies *in consequence* of his own opinion of his danger, as the following cases will show. It is remarkable how some patients get a clear impression of coming danger before the surgeon can detect symptoms of its approach.

*Case* 1. Man in good health admitted with fractured leg. There was nothing alarming, or peculiar in the case, but I found the patient one day possessed with a cool and quiet, but perfectly clear impression that he would die. Struck with his conviction, I examined his pulse and general condition minutely, without finding any ground for apprehension. I also called on Prof. Johnson, who examined him carefully with a like result. I therefore assured the patient that there was no occasion for fear. Yet within three days he was attacked with pneumonia, which brought him to the verge of the grave, and though he ultimately recovered, his presentiment came within a hair's breadth of being true.

*Case* 2. A man of apparently good constitution received a severe injury, lacerating the soft tissues extensively on the inner side of the right ankle and foot. The bones were not injured, and on examination there appeared to be nothing to discourage the hope of restoring a useful limb. He was given tincture of iron internally, and suitable local dressings, and for several days appeared to be doing well. One morning, however, as I made my rounds of the hospital, he inquired of me when I was going to amputate the limb. I was surprised at the question, but told him as I had done before, that I did not expect to do it at all, as I hoped to save it. He then assured me with perfect calmness and distinctness, that an amputation was necessary and unless performed he would die. The memory of the previous patient flashed over my mind, and I proceeded to re-examine him very closely, yet as the edges of the wounds were rosy and healthy, and the pulse good, I refused to operate. About forty-eight hours later extensive sloughing of the soft parts commenced, accompanied with such a prostration that the patient barely escaped death, and was too weak to bear an operation. The limb was now ruined, and it was only by great diligence that I succeeded in getting him slowly improved enough to bear the amputation. He ultimately recovered, with the loss of the limb at the middle of the leg.

*Case* 3. A young man entered with a varicocele. He was ruddy, and apparently healthy. I gave ether, and operated by shortening the scrotum. After the operation he took up a very clear impression that he would die. Close examination showed no reason for apprehension, yet his impression continued. After five or six days, however, my encouragements, and the assurances of the House Surgeon, seemed to convince his reason, and he gave up the idea of death. About the ninth day the wound presented a healthy, rosy aspect, and the patient was cheerful and comfortable. I therefore told him that it was not necessary to confine himself to his bed and room any more than he felt inclined. A few hours after, a nurse passing along the corridor heard a noise as of labored breathing. He entered, and found him apparently dying. The House Surgeon was summoned instantly, and found him nearly pulseless. He gave stimulants, and used artificial respiration. The pulse improved, and the patient seemed to become conscious again, but he soon relapsed, and in a few moments more was dead. The post mortem showed that the heart was healthy, but the brain displayed marks of congestion, and contained two or three clots of extravasated blood.

A moment's thought shows that the popular idea of the patient's danger being a consequence of the expectation of death, is inadmissible in all these cases. The first one took pneumonia, a disease which cannot be produced by a mere notion. The second was imperilled by extensive gangrene, which is equally remote from anything producible by fear, while the third died suddenly, after he had given up the idea of being in any danger.

---

The new amphitheatre of the Bellevue Hospital was formally opened by the Medical Board of the Hospital on Sept. 15th, 1871. The lecture-room is capable of seating eight hundred students, and is said to be the largest and most complete in the country, if not in the world. The ventilation is excellent, and the light is reflected from the rotunda.

E. W. Houghton, Esq., has given $10,000 to Dartmouth Medical College, to establish a museum of pathological anatomy.

Niemeyer's Successor.—It is said that Prof. Liebermeister, of Zurich, has been appointed to succeed the late Prof. Niemeyer.

